# Supplementary material for: Examining influential factors for acknowledgements classification using supervised learning
Source: PLoS One. 2020 Feb 14;15(2):e0228928. doi: 10.1371/journal.pone.0228928 (PMC7021295; doi:10.1371/journal.pone.0228928)
Supplement: S1 File — The categories used in the experiment, also can be found at https://doi.org/10.6084/m9.figshare.11302280. (PDF) [file pone.0228928.s001.pdf]

Supporting Information 1. The acknowledgement categories

To classify acknowledgement sentences manually, we provided the detail of our rules in this guideline, which describes the categories with an example as well as decision aids in semantically ambiguous cases.

Categories	Descriptions	Reference sources	Example of sentences
**Peer interactive communication and technical support**	Acknowledge people or organizations for advising and supporting during processing research and reporting the study as a manuscript.<br><br>This category represents the specific suggestions or information as well as support in the technical and analysis in the research, such as studying design and analysis tools, providing help in the use of laboratory tools, allowing use of the study area, such as land, plant, and equipment, and sample preparation.<br><br>This category also covers making a discussion, comment, and assessment on the study and the report as a manuscript by reviewing, editing, proofreading, and linguistic supporting.	McCain (1991),<br><br>Cronin and Overfelt (1994),<br><br>Cronin (1995),<br><br>Cronin, Shaw, and La Barre (2003),<br><br>Khabsa, Treeratpituk, and Giles (2012),<br><br>Díaz-Faes and Bordons (2014),<br><br>Rattan (2014),<br><br>Gurjeet (2014)	- The authors appreciate the support from the Department of Economics at the University of Zurich by *facilitating access* to Qualtrics platform.<br>- I also thank Howard Roffwarg for *sharing his recollections* with me about the origins and early days of the ontogenetic hypothesis.<br>- The authors would like to thank the Analytisch Biochemisch Laboratorium (ABL), Assen, The Netherlands for *developing a bioanalytical method*, and *analyzing betahistine* in cat blood plasma.<br>- JT, SC, SF, BP, and MR had full *access to all the data* in the study and take responsibility for the integrity of the data and the accuracy of the *data analysis*.<br>- The authors gratefully acknowledge the RADAR team of DEFRA *for their assistance with the CTS extracts*.<br>- Thanks to A. P. Mitchell for help in *the interpretation of the databases*, and R. S. Clifton-Hadley for comments on an earlier presentation of this work.<br>- We appreciate the support from the Department of Economics at the University of Zurich by *facilitating data acquisition*.<br>- We gratefully acknowledge Feng-ming Zhang from the School of the First Clinical Medical Sciences, Wenzhou Medical University, for *assistance with the experiment* and acquiring funding.<br>- Special thanks to Associate Professor Trevor Norman of the Department of Psychiatry, University of Melbourne, Austin Hospital, Heidelberg, VIC, Australia for the *provision of equipment required for behavioral testing*.<br>- We thank Dr. Michael Nitsche for *assistance with developing our MEP protocol*.<br>- Manfred Herrmann for *providing a working space and an examination room*.<br>- Thanks to Durland Fish and Peter Krause for *sharing laboratory space and earlier discussions* of the project.<br>- The authors thank Dr. Takatoshi Mochizuki for his *technical support with the sleep scoring software* and for his *valuable suggestions* for this *manuscript*.<br>- We would like to thank the department of rehabilitation center, children's hospital of Chongqing medical university for *recruiting infants* and their help in *data collection*.<br>- The authors are grateful to Dr. Andrew Liu, Memphis, for the *pLV7Bmal plasmid* and to Dr. Stephen Brown, Zürich, for *providing the plasmids pMD2*.<br>- We thank Dr. Bronwyn Gaut for her *critical reading of the manuscript*.<br>- We thank Colin Smith for his *insightful comments* in reviewing the manuscript.

	**<u>The example of keywords and patterns in the sentences.</u>**<br><br>Discussion<br>Comment<br>Suggestion<br>Advise<br>Guidance<br>Early stages<br>Inspired discussions<br>Constructive comments<br>Helpful discussions<br>Helpful suggestions<br>Earlier comments<br>Fruitful discussions<br>Useful discussion<br>Insightful discussion<br>Critical comments<br>Editorial<br>Editing<br>Editor<br>Reviewer<br>Anonymous reviewer<br>Editorial view<br>….<br>Statistical analysis<br>Data analysis<br>Histological analysis<br>Chemical analysis<br>Meta-analysis<br>Image analysis<br>Microarray analysis<br>MS analysis<br>Literature analysis<br>Molecular analysis<br>Gene analysis<br>Genetic analysis<br>Structure analysis		- The authors thank the Washington Department of Fish and Game *for use of land and resources for the field experiment*; the USDA Agricultural Research Station for greenhouse space.<br>- AC was *responsible for* statistical analyses and critically revised the manuscript.<br>- ACÂ and RG were primarily *responsible for* conducting analyses of the data and writing the manuscript.<br>- Author contributions LMT and KFC are the corresponding authors and were *responsible for* coordinating and editing the manuscript.<br>- Bi was *responsible for* the conception, design, data acquisition, analysis, drafting and revising the article, and final approval of all versions of the article.<br><br>* *responsible for*<br><br>Usually, we can find this term in the “Declaration” category, but we considered the pattern of sentence to classify. If *responsible for* is **followed by analysis or technical terms**, then we classified the sentence to be “Peer interactive communication and technical support”.

	Bioinformatics analysis<br>Mass spectrometry analysis<br>DNA analysis<br>FISH analysis<br>Phylogenetic analysis<br>FALS analysis<br>molecular analysis<br>metabolite analyses<br>spatial analysis<br>proteomics analysis<br>interpreting the analysis<br>mRNA footprinting analysis<br>Laboratory analysis<br>…<br>Collected data<br>sample collection<br>Data acquisition<br>Technical support<br>Technical assistant<br>…<br>Laboratory tools<br>Study design<br>Analysis tools<br>Designed analysis<br>Analysis software<br>Computer<br>Hardware<br>Server<br>Laboratory equipment<br>…<br>Allow<br>Access to<br>Privately owned<br>Land and resources		
**Declaration**	A declaration related to conflict of interest, the access to unpublished data, and copyright as	McCain (1991),<br><br>Cronin and Overfelt (1994),	- The research was conducted in the absence of any commercial or financial relationships that could be construed as a *potential conflict of interest*.<br>- All authors *declare* that they have no *conflict of interest*.<br>- The authors, Patrice Abry and Darryl Veitch, retain *copyright* of this code.

	well as the authority of people and organizations.<br><br>This also covers ethics approval and consent with permission to publish the data and manuscript.<br><br>This category is related to moral support and privacy concerns, as well as a non-funds declaration.<br><br>***NOTE:***<br><br>This category is the author declaration research-related information.<br><br>Hence, ***it is not*** an appreciation expression.<br><br>**The example of keywords and patterns in the sentences.**<br><br>Ethics approval and consent<br>Human subject<br>Approval<br>Ethical principles<br>Consent<br>conflicts of interest<br>Copyright	Khabsa, Treeratpituk, and Giles (2012),<br><br>Díaz-Faes and Bordons (2014),<br><br>Rattan (2014),<br><br>Gurjeet (2014)	- The U. S. Government *is authorized* to reproduce and distribute reprints for Government purposes notwithstanding any *copyright* notation herein.<br>- *Ethics approval* for analysis was also obtained from the Conjoint Health Research Ethics Board at the University of Calgary.<br>- *Ethics approval and consent* to participate Not applicable.<br>- The imaging data used in this article were *obtained from* the Alzheimer's Disease Neuroimaging Initiative (ADNI) database (*http://adni.loni.usc.edu/*); the Institutional Review Board (IRB) *approval documents* are not generally *available through this database*.<br>- Studies included within this manuscript were selected based on the quality of the work and the relevance to the topic, *and this review was not meant to be all-inclusive.*<br>- The views, opinions, assumptions and conclusions or any other information set out in this article *are solely* those of the authors and not of Novartis Foundation.<br>- The *funders had no role* in study design, data collection and analysis, decision to publish, or preparation of the manuscript.<br>- Novartis *Foundation was not involved* in the establishment of this article and this article should not be attributed to Novartis Foundation or any person connected with Novartis Foundation.<br>- The *views expressed are those of the author*(s) and not necessarily those of the NHS, the NIHR, the Department of Health or Public Health England.<br>- The present work *was performed in* fulfillment of the requirements for obtaining the degree "Dr. med."<br>- It is *currently available* on GitHub (github.com/NIF-au/TissueStack).<br>- The authors report *no disclosures*.<br>- The views and opinions expressed in this manuscript are those of the author(s) and *do not reflect official policy* or position of the Department of Defense or the U. S. Government.<br>- Any remaining *error is ours*.<br>- Acknowledgments All authors contributed equally and are *responsible for* the content and writing of this paper.<br>- Acknowledgments The authors were fully *responsible for* the content, editorial decisions, and opinions expressed in this review.<br><br>**Non-funds statement**<br><br>- ACKNOWLEDGMENT The study was done as part of the M Phil (clinical psychology) dissertation of the first author; the study was *non-funded*.<br>- Funding This is a *non-funded* study.<br>- *No sources or grants contributed* to the completion of this research.

	Authorization<br>Dedicate<br>Declare<br>Disclosure<br>Compliance<br>Disclaimer<br>Verbal consent<br>Written consent<br>Patient consent<br>Official (duty/official duties)<br>Attributed to<br>Research related-information (website/data source/source code…..)<br>Policy<br>Responsible for<br>http<br>ftp<br>www<br>Error is<br>License<br>Are solely<br>Funders had no role<br>No role<br>Was not involved in<br>Views expressed are<br>Those of the author<br>Not necessarily those of<br>Was performed in<br>Do not reflect official<br><br>- The funding agencies had no direct role...<br>- The funding agency has no involvement...<br>- The funding bodies did not have any role in…<br>- The funding body did not assist with…		

	- The funding body <u>played no role in</u>…<br>- The funding organization had <u>no role in</u>….<br>- The funding source <u>had no involvement in</u>…		
**Presentation**	The presentation of the study, findings, and a portion of the work including abstracts, posters, and oral presentations in conference, proceeding, or seminar.<br><br>**<u>The example of keywords and patterns in the sentences.</u>**<br><br>Presentation<br>Present<br>Poster<br>Oral<br>Conference<br>Proceeding<br>Seminar<br>Annual<br>Symposium<br>Annual meeting<br>Present …congress<br><br>***NOTE:***<br><br>This should be a simple statement regarding	To the best of our knowledge, this category is not mentioned in the previous studies.<br><br>However, where we investigated the acknowledgement pattern in our raw data, the presentation statements are shown in this section.	- Preliminary *versions of these data were published in* abstract communication and *poster presentation* at the European Dialysis and Transplant Association (EDTA) *conference,* June 3–6, 2017 in Madrid, Spain.<br>- The abstract of this paper was presented at the American Society of Hematology *annual meeting*, December 6–9, 2014, San Francisco, CA, USA, as an *oral presentation*: http://www.bloodjournal.org/content/124/21/202.<br>- This research *was presented* at the Society for Neuroscience (SFN) *Annual Meeting*, San Diego, CA, USA, 9–13 November 2013.<br>- A preliminary analysis of the data reported in this work was *presented* in condensed form at the XII SIAMOC *Conference* and at the XVI International Congress of Parkinson’s Disease and Movement Disorders.<br>- This work *was presented as a poster* abstract *presentation* at the Neurocritical Care Society Meeting on October 11, 2017.

	presentation at a conference.<br><br>Normally, ***it is not*** an appreciation expression.		
**Financial**	Grant support information on research. The acknowledgment and declaration of grants and scholarships received by the researcher from external or internal funding.<br><br>**<u>The example of keywords and patterns in the sentences.</u>**<br><br>The keywords usually co-occur with ORGANIZATION and SUPPORT.<br><br>Funded by<br>Supported by<br>Financial support<br>Personal fee<br>Funding from<br>Fund in part by<br>Fund by grant from<br>Finance by<br>Project conduct<br>Payment from<br>Grant from<br>Co-fund by<br>Funding provided by	Cronin and Overfelt (1994),<br><br>Diaz-Faes and Bordons (2014),<br><br>Giles, Councill, and Gray (2004),<br><br>Rattan (2014),<br><br>Khabsa, Treeratpituk, and Giles (2012),<br><br>Nadine, Adele, and Jen (2017),<br><br>Gurgeet (2014),<br><br>Cronin, Shaw, and La Barre (2003),<br><br>Cronin (1995)	- The authors thank the technology transfer office of the University of Geneva (UNITEC) for their *financial support* for the development of the technical interface to couple the gyroscope to the cochlear implant processor through the INNOGAP proof-of-concept *fund*.<br>- S. B. *was supported* in part by the ERC advanced *grant* 268540-PBDR.<br>- This work *was funded* by two NIH MIDAS grants (U01GM110712 and 5U54GM111274) and a WHO *grant* 353558 TSA 2014/485861-0.<br>- D. A. *was supported* by the Medical Research Council (U105260566) and Public Health England.<br>- We acknowledge *support by* the DFG Open Access Publication Funds of the Ruhr-University Bochum.<br>- The data used in this report has been collected with *the support of* a variety of *grants* and *salary support* over the years.<br>- Acknowledgments This work *was funded* by Zoetis.<br>- This research was part of a master thesis *funded by* CAPES (Coordination for the Improvement of Higher Education Personnel).<br><br>***NOTE:***<br><br>1) Usually, we classify the sentences that consist of a "declare" term in the "Declaration" category, but for *grant declaration*, we classified as *"Financial"*.<br><br>- Competing interestsThe author *declares* that he has *received honoraria* for advisory board and steering group meetings, lectures, and travel expenses from Abbott, Baxter, BBraun, Danone-Nutricia, Fresenius Kabi, Lyric, and Nestle-Novartis.<br>- Conflict of interest statement: DS *declares* *honoraria* from Bristol-Myers Squibb, Merck Serono, Sysmex, Amgen, GrÃ¼nenthal Group, and Immunocore; and has *received research funding* from Bristol-Myers Squibb.<br>- GS also *declare* to have *received support* for travel to meetings for the study or other purposes from GlaxoSmithKline Biologicals SA.<br><br>2) The sentences which consist the terms of "the recipient of", "fellowship", and "Fellow of" , and <u>follow by foundation organization or grant number</u>, then we classified to "*Financial*", otherwise, we classified to "General statement"

	Invest in<br>Support in part by grant<br>Funds<br>Scholarships<br>Agency<br>Fellowship<br>Financial disclosure<br>Funding source<br>Funding statement<br>Received payment<br>Salaries<br>Personal fees<br>Under contract<br>To acknowledge support from…		- Nuno Henrique Franco is *the recipient of* a post-doctoral grant (reference SFRH/BPD/85978/2012) by the Portuguese Foundation for Science and Technology (FCT).<br>- CS is a EMBO Long-term *fellowship* holder (ALTF 587-2016).<br>- Olivier Neyrolles is *a fellow* of a CNRS-ATIP *fellowship*.
**General acknowledgement**	The authors express appreciation to people or organizations that offered encouragement during the study.<br><br>This acknowledgement is not appreciation for specific assistance. Therefore, it usually mentions organizations (e.g., research center, university, and laboratory) and persons (a member of a research center, participants: interviewers, interviewees, and participants).<br><br>**The example of keywords and patterns in the sentences.**	Cronin and Overfelt (1994),<br><br>Giles, Council, and Gray (2004),<br><br>Ratten (2014),<br><br>Nadine, Adele, and Jen (2017),<br><br>Gurjeet (2014),<br><br>Cronin, Shaw, and La Barre (2003),<br><br>Cronin (1995)	- The authors also *thank* the *patients and their families* for informed consent.<br>- Special *thanks to* Fay Horak and OHSU's Neurology Department *for their support*.<br>- We are *grateful to* Dong.<br>- The authors are *grateful to* all participating patients and their families.<br>- We would like to *acknowledge the contributions* of all personnel at the neurosurgical research laboratory (R2:02), Department of Clinical Neuroscience, located at Neurocentrum, Karolinska University Hospital, Stockholm, Sweden.<br>- The authors *thank the whole staff* from the Neurology, Radiodiagnostic and Nuclear Medicine departments, the certified neurologist EEG, the University Hospital of Liège and the patient.<br>- We are most *grateful to all physicians* participating in the study.

	Research center<br>University<br>Laboratory<br>Lab<br>Participants<br>Volunteers<br>Patients<br>Participated<br>Interviewer…thank<br>Interviewee…thank<br>Their families…thank<br>Thank…contribution		
**General statement**	The general statement presents the information that is not related to a study directly—for example, the person or institute name information explanation. This category is a kind of ***unclassified*** category.<br><br>***NOTE:***<br><br>This category is the author's general statement. Hence, ***it is not*** an appreciation expression.<br><br>**Pattern of sentence**<br><br>Is a…. (no thanks)<br><br>Authors' information	Rattan, (2014),<br><br>Gurjeet (2014)	- Within HSS, Tzipora *Kuba's* research management was necessary for the successful completion of this project.<br>- Nicolas Morin held a professional health care studentship from the Fonds de la Recherche en Santé du Québec.<br>- Thiago J. R. Rezende: none.<br>- Daniel P. Cardinali *is a Research Career Awardee* from the Argentine Research Council (CONICET) and Professor Emeritus, University of Buenos Aires.<br>- Vesna Jevtovic-Todorovic *was an* Established Investigator of the American Heart Association.<br>- CZ *is a* veski innovation fellow.<br>- AB *is a* KUL post-doctoral fellow.
